# Recruitment problems in psychosocial oncology research

**DOI:** 10.1002/pon.4792

**Published:** 2018-06-29

**Authors:** Jacques J.D.M. van Lankveld, Joke Fleer, Maya J. Schroevers, Robbert Sanderman, Brenda L. den Oudsten, Joost Dekker

**Affiliations:** ^1^ Faculty of Psychology and Educational Science Open University of the Netherlands Heerlen the Netherlands; ^2^ Department of Health Psychology University of Groningen, University Medical Center Groningen Groningen the Netherlands; ^3^ Department of Medical and Clinical Psychology Tilburg University Tilburg the Netherlands; ^4^ Department of Psychiatry, Amsterdam Public Health Research Institute VU University Medical Center Amsterdam the Netherlands

**Keywords:** patient recruitment problems, psychosocial cancer research, adaptive distress, maladaptive distress, desire for help

**Key Points**

- Patient‐reported distress does not necessarily reflect the patient's actual desire for help.
- Distress may be either adaptive and helpful, or maladaptive.
- Feasibility studies should assess interest and willingness of patients to receive professional psychosocial care.
- Research on optimal recruitment strategies will improve enrollment in patient‐based research in psychosocial oncology.
- Greater reporting of problems with patient recruitment in clinical outcome studies in the field of psychosocial cancer treatment will help minimizing publication bias.

In our professional experience with conducting psychosocial cancer research, we have all experienced problems with recruiting cancer patients and survivors for participation in outcome research. As researchers, we work in a highly competitive environment in which many compete for scarce resources. We sometimes end up submitting ambitious grant proposals whose feasibility, in patient recruitment, may be less than optimal. For instance, in 2013, both van Lankveld and colleagues and Schroevers and Fleer and colleagues received funding for innovative and methodologically rigorous (ie, following Consolidated Standards of Reporting Trials criteria) intervention studies. In both studies, the inclusion rate was disappointingly low (both lower than 1%), even though power calculations were based on prevalence rates of psychosocial problems reported in the recent literature. Similar problems are probably experienced by many fellow researchers, and have also been reported or hinted at in some publications, eg, Fredman^1^ and van Scheppingen.^2^ Our goal here is to make a plea for more extensive reporting about this issue, both in the interest of minimizing the risk of publication bias in the field of psychosocial oncology in case study results would remain unpublished, and to allow colleagues to learn from these negative experiences, and thus to avoid wasting additional investigational resources.

We believe that many of the problems experienced in recruiting patients into our studies reflect a lack of awareness among researchers of barriers to recruitment, that result from a mismatch between patient characteristics, the types of intervention that we offer, and the ways we select and approach potential research participants. Patient characteristics include, for example, their felt desire for help, personality characteristics, and their preference for specific treatment type. Types of intervention include, for example, individual vs group treatment and cognitive behavioral vs supportive‐expressive approaches. We are convinced that both methodological and patient‐related factors contribute here.

Methodological factors include the use of screening instruments that have low predictive validity and do not succeed in identifying those patients who desire supportive psychosocial care, as well as inefficient procedures for approaching cancer survivors. An example of the first factor was provided by van Scheppingen and colleagues^2^ who conducted an RCT investigating the efficacy of problem‐solving therapy for cancer survivors. They reported that screening based on symptom checklist scores and 1 additional question on need for services proved unsuccessful for recruiting sufficient cancer patients in need of care. An example of the impact of procedural characteristics on recruitment rates was published by Fredman and coworkers.^1^ They investigated a couple‐based intervention aimed at relational enhancement for breast cancer survivors. Couples were more likely to participate when they were contacted at home or at a follow‐up appointment at the cancer clinic, compared to when they were asked to participate when first diagnosed. Shorter geographical distance between home and treatment facility location also increased the likelihood of participation. Another example of the latter point was given by Rabin and colleagues.^3^ They observed significant differences among young adult cancer survivors in enrollment rates for an exercise intervention study between personal recruitment at a clinical facility and at cancer‐related social events, and when using nonpersonal procedures via postal mail, telephone, advertisements on the internet, radio, television, social media, or other means of contact. Recruitment including personal contact in an oncology clinic yielded the highest inclusion rates, although the cost‐effectiveness of nonpersonal recruitment strategies was higher.

Patient‐related factors are also related to diminished study participation in psychosocial oncology research, including patient characteristics, such as both younger and older age,^3^ and the perceived magnitude of burden associated with study participation. Another important factor that may account for low inclusion rates has to do with the lower than expected desire for care in patients, despite high distress scores. Screening for inclusion eligibility in an investigational treatment based on distress level is common practice and is included in several guidelines for clinical practice, eg, Network.^4^ You should only include patients in an intervention study when screening has shown that their distress levels are so high that improvement from an intervention can be expected. However, elevated distress does not necessarily coincide with patient‐perceived wish for help, nor with the desire of patients for referral to psychological or psychosocial treatment.^5^, ^6^ Thus, instruments that are recommended for distress assessment in psychosocial oncology, including the Distress Thermometer and the Hospital Anxiety and Depression Scale, may not be adequate for detecting desire for care. In sum, both symptom level and emotional distress level are probably insufficient to identify the actual desire for help and willingness to participate in intervention research.

A factor that may play a role here is the dynamic coping strategy of patients and their family. Coping involves the ways that patients deal with factors such as stigma associated with cancer and with the use of mental health care, and with the distress experienced during the diagnostic phase and during primary cancer treatment. Some patients may already have sought professional help when they are invited for study participation; others may be convinced that the distress will go away without professional intervention, or with the help of friends and loved ones. Patients sometimes report that they want to move on with their lives, and not focus on their difficulties. Patients find some cancer‐related problems more difficult to discuss with their health care provider than other problems. For instance, studies addressing problems with sexual functioning and low sexual satisfaction as a result of cancer and cancer treatment have met with low participant rates,^7^ possibly because discussing sexual health is a sensitive topic for both patients and health professionals or because it may not be a high priority issue for the patient at that time. Thus, more research is needed to investigate patients' reasons for reporting (absence of) a wish for care, as well as the contributions to these reasons of factors such as (inadequate) problem recognition, knowledge and beliefs, coping self‐efficacy, and social support. Such research provides insight into which factors need to be targeted to assist patients in making an informed decision about care uptake.

A possible explanation for the discrepancy between distress and service uptake is that the presumed link between cancer‐related distress and patients' desire for professional help is not based on emotion theory and the relationship between emotion and psychopathology.^8^ Dekker and colleagues argued that it is not the intensity but the nature of emotional responses which determines whether emotions are maladaptive. Using the results of research on the relationship between emotion dynamics and psychopathology,^9^ they argued that emotional responses, reflected in elevated distress scores, often serve an adaptive purpose. Emotional reactions to cancer and cancer treatment only become maladaptive when they linger and perpetuate over time, or when they are extreme and unstable.^9^ Emotional responses may also become maladaptive when they hinder adaptive coping. Dekker and colleagues^8^ further argued that patients' need for psychosocial care depends on whether emotional responses are adaptive or maladaptive. Patients who experience maladaptive emotional responses seem to be in need of professional mental health care, defined as psychotherapy, pharmacotherapy, or emergency psychiatric care. In contrast, patients who experience adaptive emotional responses do not seem to be in need of professional mental health care. Instead, they may be primarily in need of support from relatives, friends, and primary caregivers (ie, doctors and nurses) and low‐intensity psychosocial interventions. Further research on the distinction between adaptive and maladaptive emotional responses and on tailoring of interventions to the needs of patients with cancer is needed. Such research has now begun to emerge.^5^

In conclusion, improving the recruitment of patients to psychosocial intervention studies that target emotional needs can be facilitated by carrying out feasibility studies that assess both the prevalence of problems in the target population and the interest in and willingness of patients to receive professional psychosocial care. Estimates of perceived symptom burden and functional impairment alone can result in serious overestimates of uptake in psychosocial intervention studies.

## CONFLICT OF INTEREST

None of the authors have received financial support for the preparation of the manuscript or have any other conflict of interest to disclose.
